# Integrating qualitative insights with large secondary data: a protocol for a community-engaged mixed-methods study on adolescent substance use

**DOI:** 10.3389/fpubh.2025.1664492

**Published:** 2025-11-12

**Authors:** Kazi Priyanka Silmi, Paris Adkins-Jackson, Blanca Meléndrez, Nghi Dang, Segen Zeray, Carlos Cardenas-Iniguez, Erika Pugh, Yailene Perez, Nayeli Cervantes, Precious Peters, Stephanie Hammonds, Igdalia Covarrubias Reyna, Delfina Álvarez, Maribel Arias, Jovita Murillo, Marybel Gonzalez

**Affiliations:** 1 Department of Psychiatry and Behavioral Health, College of Medicine, Ohio State University, Columbus, OH, United States; 2 Departments of Epidemiology and Sociomedical Sciences, Columbia University Mailman School of Public Health, New York, NY, United States; 3 Center for Community Health, Altman Clinical and Translational Research Institute, University of California San Diego, San Diego, CA, United States; 4 Keck School of Medicine, University of Southern California, Los Angeles, CA, United States; 5 Comité Organizador Latino de City Heights (COLCH), San Diego, CA, United States; 6 School of Public Health and Information Sciences, University of Louisville, Louisville, KY, United States

**Keywords:** community-engaged research, mixed-methods design, secondary data, adolescent substance use, qualitative

## Abstract

Understanding the factors of risk and resilience for youth substance use requires interdisciplinary and multi-level methodological approaches that integrate the community of study into the research process. This protocol describes a novel, community-engaged, modified convergent mixed-methods design to investigate factors of neighborhood social risk and resilience (NSRR) in relation to substance use and neurocognition among Hispanic adolescents living in neighborhoods with unequal opportunities and restricted access to resources. We propose a design for integrating primary qualitative data with secondary data from the Adolescent Brain Cognitive Development (ABCD) Study, the largest longitudinal adolescent cohort in the United States. Guided by community-engaged research practices, and socio-ecological and health disparities frameworks, the protocol centers on the experience of young adolescents. Our design prioritizes partnerships among academic, community, and grassroots organizations to co-develop study design conceptualization, recruitment and analysis plan, along with the interpretation and dissemination of results. The secondary quantitative data analysis leverages advanced statistical modeling to examine relationships between neighborhood level factors and substance use, providing measurable insights both at the population level and at the neighborhood level. Qualitative interviews with adolescents provide an opportunity for collecting a rich, community-grounded perspective that captures the lived experience of adolescents in how neighborhood factors shape adolescent health behaviors. Findings will be synthesized using data integration and shared through academic, community-facing, and policy channels. This protocol highlights the importance of a community-engaged mixed-methods design that strengthens the cultural relevance, actionability, and validity of adolescent substance use research by embedding community voices throughout all phases of the research process.

## 1 Introduction

In substance use (SU) research, community-engaged research (CEnR) is increasingly recognized as central to championing health opportunities for all as it incorporates voices from those with differing viewpoints and lived experience (1–3). CEnR, broadly defined as meaningful engagement between researchers and community members throughout the research process, enhances cultural relevance, trust, and the interpretive depth of findings (2, 3), thereby increasing the validity, actionability, and impact of the research findings. Similarly, mixed-methods design supports more reliable findings by combining the strengths of quantitative methods to quantify prevalence, patterns, or mechanisms of behavior along with qualitative methods to contextualize the findings through community-informed narratives that illuminate structural and sociocultural influences (4, 5). Mixed methods studies using large-scale secondary datasets can save time and costs, allowing researchers to efficiently produce findings that can inform public health programming and policies (6, 7). A community-engaged mixed-methods design that incorporates primary qualitative insights and community inputs to conduct and interpret findings from secondary data analyses has the potential to leverage the strengths and mitigate the limitations of each research method and data source type.

CEnR can advance the understanding of mechanisms that contribute to disparities in health outcomes by grounding findings in the lived experiences of adolescents of all backgrounds (8). Hispanic adolescents account for one fourth of the American youth population but may lack equal opportunities that promote resiliency to SU prevention and well-being across their lifetime (9). A recent study suggests that Hispanic adolescents are significantly more likely to initiate SU before the age of 13 in comparison to non-Hispanic adolescents (10), which may be attributed to experiencing adverse structural systems and social experiences that influences health. Understanding SU among Hispanic adolescents requires a holistic, integrative, and multilevel approach to address the unique needs of those affected. Structural and social factors are often shaped by environmental and sociocultural factors identified in the National Institute on Minority Health and Health Disparities (NIMHD) Research Framework (11, 13). These factors are not isolated or unidimensional but are multiple, intersecting, and reinforcing each other across layered system levels, from the micro to the macro. Every socioecological level, starting from individual to societal, influences the lived experiences, access (or lack thereof) of resources, and opportunities available to Hispanic adolescents. This can either reduce or increase their likelihood of SU initiation, experimentation, regular use, risky use, dependence, addiction, and crisis. A scoping review on alcohol and tobacco use among Hispanic adolescents found that most published studies focused on sociocultural domains within individual and interpersonal levels (12), highlighting the need for research that investigates broader social and structural determinants like neighborhood-level risk and resilience factors.

Large-scale secondary datasets provide valuable opportunities to examine patterns and multi-level predictors of SU among youth (7). To enhance their utility, these datasets can be complemented with community-based data collection efforts that capture the nuanced context, specific resilience factors from all backgrounds, and structural challenges uniquely experienced by adolescents with a wide variety of lived experiences (6, 8). Without community input, there is a risk of reinforcing deficit-based assumptions or conflating individual-level characteristics with structural-level root causes (13, 14). Secondary datasets are broadly used in youth SU research for longitudinal tracking, cross-site comparisons, and advanced statistical modeling across large and diverse samples. These, often publicly available datasets, can facilitate replication, generalizability across populations, and interdisciplinary research.

In public health research, big data is increasingly utilized to investigate trends in SU and associated risk and protective factors, related real-world insights into population outcomes and public health prevention and response efforts (15, 16). However, analysis done with secondary datasets may reflect dominant cultural norms and omit context-specific risk factors, culturally specific protective factors, community assets, and experiences of systemic disadvantage. To address this, studies can integrate the CEnR approach for contextual community insights with mixed-methods design that combine the statistical power of large datasets with the depth of qualitative inquiry (17, 18). The Adolescent Brain Cognitive Development® (ABCD) Study is the largest long-term study on youth health in the United States, with a primary focus on understanding risk and resilience for emergence of SU during adolescence. Almost one in four adolescents in the ABCD Study are Hispanic (19).

In this paper, we describe a community-engaged mixed-methods study that integrates secondary quantitative data from the ABCD dataset with primary qualitative interviews conducted with adolescents. We highlight the importance of implementing CEnR approaches across all phases of the research process, including shared decision-making and iterative feedback loops. We describe our ongoing experience of engaging with community partners through the different processes of study conceptualization to research data collection and analysis to the interpretation of both qualitative and quantitative findings. Finally, we describe how we plan for the CEnR approach to shape the synthesis and dissemination of results to enhance their relevance, accuracy, and impact, along with nurturing sustainable, mutually beneficial academic-community partnership.

## 2 Study aims and research questions

To guide our research aims, we utilized a socio-ecological framework of adolescent development (13). This socio-ecological framework of youth health draws from the NIMHD Research framework and Bronfenbrenner’s Ecological Systems Theory and centers strength-based and community-centered approaches for scientific inquiry of risk and resilience for adolescent health (11, 13, 20). This multilevel conceptual model provides recommendations for conducting rigorous health disparities research that engages community experts through all levels of the socioecological framework via different research stages (study conceptualization, recruitment, analysis, interpretation, and dissemination) to advance equal opportunity in both scientific knowledge and public health outcomes.

**Figure 1:**
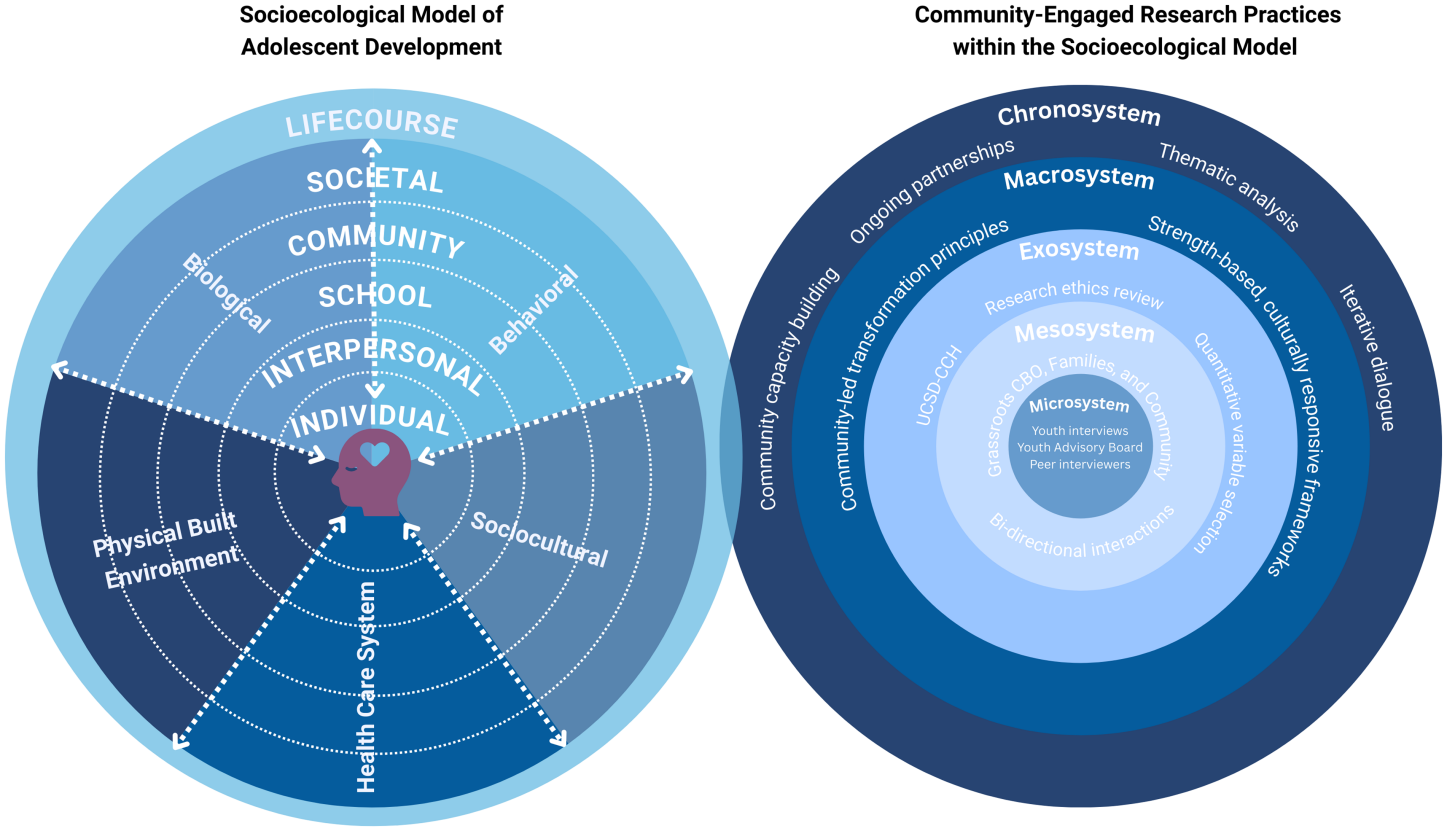
Multilevel CEnR approaches at levels of the socioecological frameworks. Adapted from “Our adaptation of the NIMHD health disparities research framework consists of a conceptual model with five domains for the social determinants of health: biological, behavioral, sociocultural, physical/built environment, and the healthcare system, each spanning five contextual levels important for adolescent health: individual, interpersonal, school, community, and societal contexts, with such early exposures having an impact across the life course” by Gonzalez et al., licensed under CC BY-NC.

In line with this framework, we use a community-engaged research, convergent mixed-methods design, to examine the influence of neighborhood social risk and resilience (NSRR) on neurocognition and adolescent SU. The quantitative component leverages the ABCD dataset to assess the influence of NSRR conditions on adolescents’ decision-making and SU. To complement this analysis, a qualitative component is introduced to explore community and adolescent perspectives about the NSRR influences on adolescent well-being and SU. Figure 1 illustrates how we used multilevel CEnR approaches at levels of the socioecological frameworks.

This study protocol describes the goal of our study, with particular emphasis on the integration of CeNR approach in our research design and methodology by explaining the (i) selecting, analyzing, and interpreting quantitative variables derived from a large secondary dataset; (ii) co-developing the qualitative interview guide, recruiting participants, and fostering sustainable community partnerships; and (iii) engaging in iterative dialogue with community stakeholders to interpret and integrate findings across data sources in a strength-based, contextually grounded manner that informs future research and intervention strategies.

## 3 Methods

### 3.1 Study overview: mixed-methods study design

This study employs mixed methods convergent, parallel design combining a primary qualitative investigation with a secondary, large-scale quantitative analysis from an existing dataset (21, 22). Although the secondary quantitative data were collected prior to the qualitative strand, our analytic work with these data occurred convergently with the collection and analysis of qualitative data with the intent to integrate the results. Consistent with the guidance, both data components were analyzed separately. The results will then be reviewed, compared, and integrated to generate insights on risk and resilience factors influencing substance use and well-being among adolescents living in neighborhoods with restricted and limited access resources.

The mixed-methods approach improves the rigor of the research by integrating data from two modalities to enhance our understanding of a phenomenon. The mixed-methods approach in our study aims to (i) integrate findings from qualitative and quantitative research methods to leverage the strength of both types of methodologies and (ii) incorporate a more expansive range of perspectives by centering the adolescent experience to understand complex mechanisms that impact adolescent SU and well-being (23).

As a commitment to the best practices for health disparity research, we applied a CEnR approach to foster meaningful partnership that shares responsibilities and mutual benefits among researchers and community members (2, 3, 24). We implemented the Community-Led Transformation (CLT) principles, which recommend that equitable community-academic partnerships should be: (i) community-led, (ii) co-designed, (iii) trust-driven and partnership-based, (iv) embodying cultural humility, (v) healing-centered and trauma-informed, (vi) holistic and strength-based, (vii) adaptive and responsive, (viii) grounded in shared funding, and (ix) sustainable (25).

**Figure 2:**
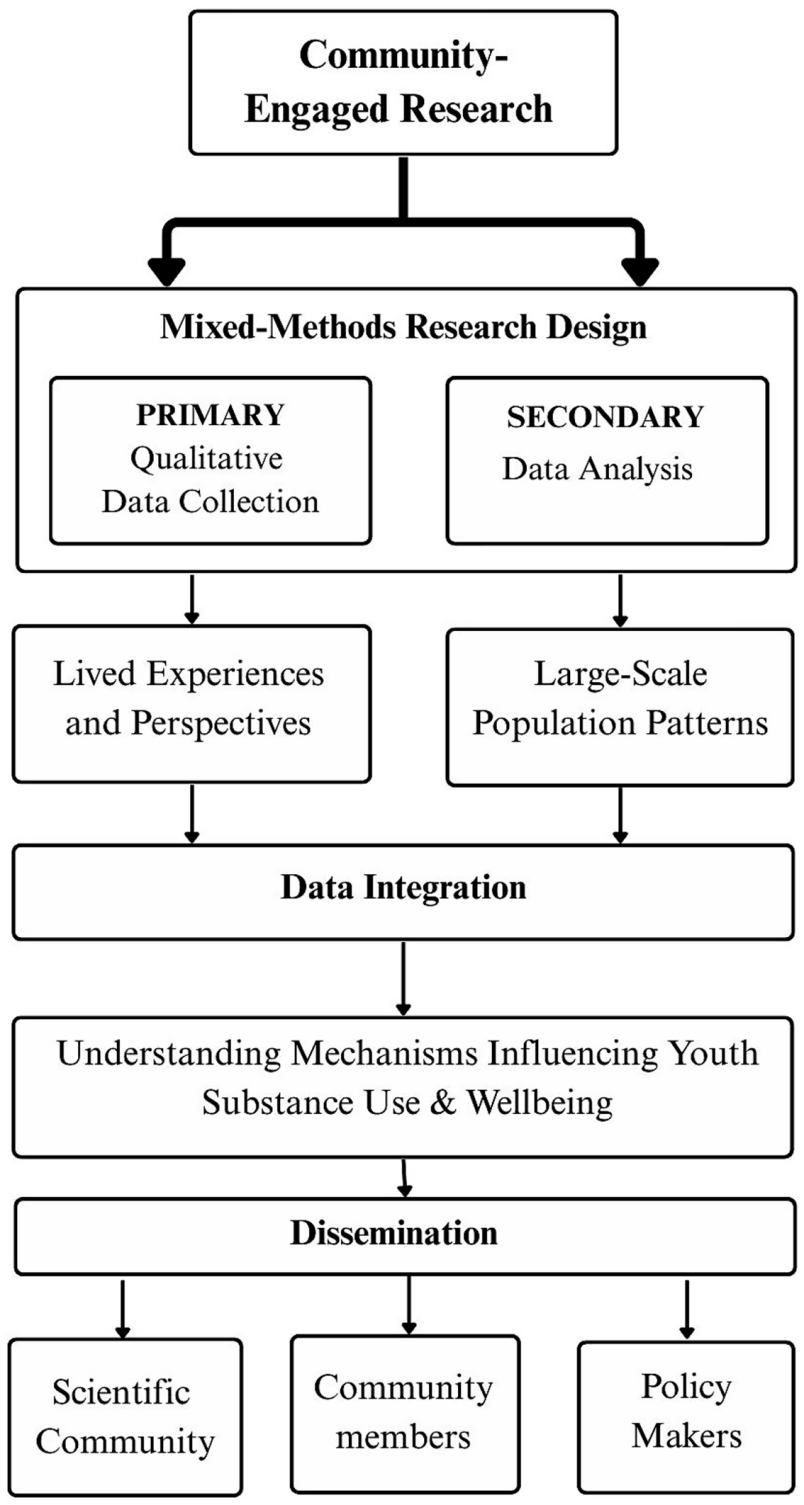
A community-engaged mixed-methods design integrating secondary data with primary qualitative findings.

A visual representation of the study design can be found in Figure 2.

### 3.2 Procedure

#### 3.2.1 Quantitative secondary data source

The ABCD Study is a large-scale, longitudinal research initiative that follows 11,880 adolescents, recruited at ages 9–10 from 21 study sites across the United States (19, 26). Applying an epidemiologically guided recruitment strategy, primarily reaching out to schools and community partners in the catchment areas representing the demographics of the national census data for the recruitment regions, the participants aged 9–10 years of age were recruited between 2016 and 2018. The ABCD cohort is composed of 48% females, 50.7% White, 25.1% Hispanic, 14.5% non-Hispanic Black, 5.0% Asian, and 4.7% identifying as non-White other races. Additionally, 53% of primary caregivers held a bachelor’s degree. All participants are invited to participate in bi-annual (phone interviews) and annual in-person/hybrid visits. Retention of participants in ABCD has been excellent, with completion rates of 95% or above at annual visits.

The study collects annual data from both youth and their families across a comprehensive range of domains, including neurocognitive, behavioral, environmental, and health-related variables. In addition to individual and interpersonal-level data, the ABCD Study incorporates information about participants’ structural environments (both social and physical) through external datasets linked via geocoding of residential addresses (14, 27). This integration of area-level indicators (e.g., census tract data) enables the examination of contextual factors and their influence on developmental outcomes. In this study, we leverage this dataset to investigate how NSRR impact neurocognition and SU, particularly among a large sample of adolescents and a sub-sample of Hispanic adolescents. The large, diverse sample along with availability of a wide range of variables allowed us to test the multilevel mechanistic pathways contributing to adolescent SU in populations of all backgrounds.

##### 3.2.1.1 Key variables

Adolescent SU was assessed for alcohol, cannabis, nicotine, and 16 other drug classes using the Timeline Follow back, administered by a trained researcher (28, 29).

NSRR was assessed by the Childhood Opportunity Index (COI) 2.0 (30, 31). The COI 2.0 is derived from multiple publicly available administrative and census-type indicators that reflect neighborhood strength-based resources that facilitate social-interactive, environmental, geographic, and institutional mechanisms for healthy child development outcomes. To account for differences in measurement scales (e.g., counts, percentages, currency), raw indicator values are standardized (*z*-scores) and weighted prior to aggregation, yielding the overall COI composite score and subdomain scores. Details about the calculation of the index have been described elsewhere (30). Area-level NSRR features are shaped by neighborhood-level structural unequal access to opportunities, which consist of factors that disproportionately disadvantages neighborhoods in areas of socioeconomic conditions, environmental health, and educational opportunities (32, 33). The COI 2.0 comprises 29 indicators aggregated into an overall composite score, along with three subdomain scores: (1) Social and Economic Opportunities (e.g., poverty rate, homeownership), (2) Health and Environmental Opportunities (e.g., access to healthy food, exposure to environmental toxins), and (3) Educational Opportunities (e.g., school poverty rates, teacher experience). These dimensions offer a multidimensional view of children’s neighborhood contexts. Area-level NSRR features are shaped by neighborhood-level structural unequal access to opportunities, which consist of factors that disproportionately disadvantages neighborhoods in areas of socioeconomic conditions, environmental health, and educational opportunities (32, 33).

Neurocognition associated with the neurocognitive domain of risky decision-making was assessed using the Game of Dice Task (GDT) (34). GDT measures decision-making under explicit risks as it evaluates the influence of executive functions using a gambling procedure. Prior work has established an association between risky decision-making and SU in adolescents as well as in adults (34, 35). The participants start the game with a fictitious amount of $1,000 and may choose bets on a virtual dice roll to potentially maximize their reward. As one bets, they receive immediate feedback after each roll which may help track learning and adaptation to the consequences of choices. Safe choices include choosing options with high probability and lower reward (e.g., betting on 3–4 dice combinations) and risk choices with low probability but higher reward (e.g., betting on 1–2 dice).

##### 3.2.1.2 Data analysis

The data will be analyzed using R version 4.4.2 and RStudio 2024.12.0 (36, 37). Linear mixed-effect models will be used to examine the influence of NSRR on adolescent SU and neurocognitive markers of decision-making. In these models, NSRR will be specified as the primary independent variable, with SU outcomes and neurocognitive measure as dependent variables. Models will include (i) fixed effects (e.g., primary independent variable and relevant covariates) (ii) random intercepts specified to account for repeated measures across study waves, clustering within families, and for design site to account for site-level variability (38). We will follow the recommendations for conducting quantitative longitudinal analyses using the ABCD dataset. For more documentation on guidance on proposed analyses readers can refer to Hawes et al. (38), Thompson et al., (39), and Li et al., (40).

#### 3.2.2 Qualitative primary data collection

We conducted semi-structured interviews with 30 Hispanic adolescents to collect primary qualitative data on how neighborhood level factors influence SU in the community. We collaborated with our primary community partner, University of California San Diego Center for Community Health (UCSD-CCH) to select the study site for qualitative data collection. We identified primary data collection sites for the qualitative component of the study from neighborhoods in San Diego County characterized by low to very low COI 2.0 scores. Thus, the decision to select the study site was grounded in both community feedback and quantitative indicators of neighborhood opportunities available through the publicly available COI 2.0.

Once the study site was determined, UCSD-CCH took an active role in establishing a partnership between the researcher and the grassroots CBO, Comité Organizador Latino de City Heights (COLCH), a small non-profit organization with an established history of serving their community.

Participant recruitment for the qualitative component was conducted in partnership with a neighborhood-level grassroots CBO. This partnership significantly enhanced our understanding of the local cultural context and led to improved tailoring of the interview guide, recruitment strategies, and overall study implementation. It also laid the groundwork for potential long-term, sustainable collaboration. Additionally, the UCSD-CCH supported the establishment of a Youth Advisory Council (YAC) composed of adolescents from the local community. YAC provided valuable input on the development of the interview guide and will continue to be engaged during the interpretation phase to ensure that the findings are relevant, respectful, and grounded in lived experiences.

This study was conducted in accordance with the ethical standards of the institutional and national research committees and with the 1964 Declaration of Helsinki and its later amendments or comparable ethical standards. Ethical approval was obtained from The Ohio State University Institutional Review Board in 2024 (IRB Protocol #2023B0277).

Undergraduate research assistants with an interest in SU and equal health opportunities were recruited and trained as interviewers since they were closer to the age of the adolescent participants. The research assistants were paid and received mentorship and training in a wide variety of study relevant topics including structural determinants, adolescent health, SU, neurocognition, research methods, and research ethics.

Each semi-structured interview lasted up to 1 h (41). Prompts were used to elicit the adolescents’ perception of their neighborhood conditions and connections to adolescent health. The interview prompts were co-designed in collaboration with the Community Expert Panel to create a supportive interview environment that encourages youth participants to share their experiences openly. Details of the process and positive outcome of this co-designing are described in the Results section.

The interview guide prompted participants to reflect on their perceptions of neighborhood conditions, including what they observe daily, where teens spend their time, and which spaces are seen as safe, enjoyable, or risky. For example, participants are asked: “What do you see around you in your neighborhood?” and “What places or spaces in your neighborhood do people enjoy or like to use?” The guide also probed exploration of both positive and negative influences on youth well-being, such as supportive environments (e.g., “What are some things about your neighborhood that are good for teens?”), unsafe areas and risky activities (e.g., “What are some things about your neighborhood that might not be safe or good for teens?”), and community responses (e.g., “Have you seen any prevention efforts?”). By exploring these themes, the interviews sought to understand how neighborhood conditions shape adolescents’ experiences, exposures, and decision-making related to substance use.

Prior to participation, the interviewer obtained written informed consent from all parents or legal guardians. In addition, written assent was obtained from all adolescent participants, following age-appropriate explanation of the study procedures, risks, and benefits. All youth participants received a study-specific information sheet, which included a summary of their rights as participants, study contact information, and a list of relevant community-based resources (e.g., mental health services, crisis hotlines, and youth support programs).

Confidentiality was strictly maintained throughout the study. All data were de-identified and securely stored in encrypted, password-protected databases accessible only to authorized research personnel. Participation was voluntary, and participants were reminded of their right to withdraw from the study at any time without penalty. To protect participant privacy and confidentiality, all study findings shared during future community dissemination efforts will be presented in an aggregate and fully de-identified format. Pseudonyms will be used, and any potentially identifying information will be removed to ensure participants cannot be recognized.

##### 3.2.2.1 Data analysis

To analyze the qualitative data obtained from adolescent semi-structured interviews on how neighborhood level factors influence SU in the community, we applied a six-phase thematic analytic approach (42). This framework draws on grounded theory and the constant comparison method (43, 44), which supports iterative meaning-making across cases, where data are continuously compared to refine categories and themes (42, 43). This approach ensures that evolving patterns reflect both the uniqueness of participant narratives and emerging cross-cutting concepts.

While content analysis often involves quantifying codes and emphasizes reliability (45), we selected grounded theory analysis to preserve the richness and complexity of participants’ lived experiences. This analysis is guided by the need to produce credible, structured themes grounded in both empirical data and community context. The qualitative analytical process was carried out in five iterative phases using Dedoose, a web-based platform for qualitative data analysis. Phase 1 included coders reviewing transcripts to gain familiarity with the data. During phase 2, open coding was used to label concepts and initial themes line by line across a subset of interviews. Each transcript was coded independently by at least two coders, who subsequently met to discuss agreements and discrepancies. The codes were based both on emergent language from the participants and deductive codes informed by prior literature on youth substance use, prevention engagement, and neighborhood risk. The constant comparison technique supports theme identification through iterative comparisons across cases (46) and is a core analytic strategy in qualitative research for ensuring conceptual coherence (47). This method guided the development of code definitions and the merging of overlapping codes. In phase 3, coders engaged in axial coding, a process of linking categories and concepts by identifying relationships among them such as causal conditions, phenomena, context, intervening conditions, actions/interactions, and consequences. In phase 4, axial themes were synthesized into core categories. Using selective coding, the team consolidated overlapping codes, refined definitions, and developed a codebook that unified the emerging thematic structure (47). Constant comparison continued through this phase to ensure clarity and alignment across all coders. Interrater reliability was assessed across six transcripts (~20% of the total sample) to establish agreement before moving forward. Phase 5 focused on theme extraction and definition. Themes were entered into a shared matrix to support identification of patterns across cases and coders. Coders compared excerpts and groupings of codes to define overarching themes, ensuring both convergence and diversity across youth narratives. The themes were then clearly defined, labeled, and documented, including sub themes where applicable. The coders used maintained analytic memos while coding, which were shared during weekly meetings.

The summary of themes will be done in service of preserving the lived experiences of participants (i.e., preserving sentiments and voices), and information is organized for future meaningful use by researchers and stakeholders. Findings will be reported in manuscripts for peer review, and a separate summary report will be prepared for community members.

#### 3.2.3 Mixed-methods findings: data integration

Data integration of our mixed methods findings will take place at the interpretation and reporting level, after each of the qualitative and quantitative strands are first examined, explored, and interpreted separately via independent data analysis or separative approach (48). Prior to starting the data integration process, we will make sure that data from each method has been analyzed thoroughly, so that the results can be presented in a way that clearly depicts the findings from each individual method/data source as well as the integrated contribution to address the research aims. We will integrate the quantitative and qualitative findings using two complementary strategies: (i) integration through narrative, in which findings from both strands are woven together and presented using a side-by-side comparison to directly compare and contrast patterns; and (ii) integration through joint displays, which will visually align results from both strands to highlight convergence, complementarity, expansion, and/or divergence (48, 49).

Through a convergent parallel mixed methods design employing data collected from two different sources and methodologies, we aim to compare and contrast findings from (i) primary qualitative data representing first-person testimonials of the lived experience of Hispanic adolescents about how NSRR may influence decisions to engage with SU and (ii) quantitative findings from secondary dataset of a large sample of adolescents to examine the same question more broadly using relevant available variables, and (iii) integrate findings from both strands to strengthen validity through methodological integration, which is the use of multiple methods to study the same research question, thereby enhancing the credibility of each strand’s findings. This approach is particularly valuable in health disparities research, as it allows for a multidimensional integration of complex, context-dependent experiences such as adolescent SU (50, 51). We hypothesize that data integration will reveal convergence between youth-reported NSRR and those that predict neurocognitive and SU outcomes in the large, representative ABCD dataset. By comparing these two sources of lived experience, one derived from narrative accounts and the other from population-level data, we will generate insights on how NSRR influences SU and cognitive development in youth that is likely to extend beyond the scope of either method alone.

To ensure that research findings are meaningful and actionable, we have begun and have planned data dissemination sessions with community stakeholders to collaboratively examine areas of convergence, complimentary, or divergence across qualitative and quantitative data. This participatory process acknowledges the value of community expertise in interpreting complex data and enhances the credibility and relevance of findings for local contexts. Engaging stakeholders in this way is especially important when integrated data yields mixed results, as it facilitates transparent dialogue about differing interpretations and supports informed, community-grounded decision-making (52).

### 3.3 CEnR in practice

Inspired by the CLT principles, our CeNR strategy prioritizes long-term, trust-driven partnerships that center community voice, foster cultural humility, support sustainable collaboration, and build community capacity. This commitment extends beyond data collection to ensure long-term value for participating communities. To strengthen our academic-community partnerships, we provided community research literacy training, health education workshops, and research advocacy tools. Additionally, we established systems of continuous feedback loops and support for future community-led research. These key strategies are further discussed below. This inclusive and collaborative approach ensures that both the research process and its outcomes are culturally grounded, contextually relevant, and aligned with the values and priorities of the communities involved.

Our CEnR approach has been an iterative process that occurs with multiple community stakeholders through all levels and phases of research. Engagement began during study conception at the proposal stage with a core community partner organization and expanded post-funding to include specific local community stakeholders. These partners have been key in the research process by contributing to variable selection for the quantitative phase, co-developing qualitative interview guides, refining recruitment and compensation strategies, and participating in iterative interpretation and dissemination planning. This inclusive and collaborative approach ensures that both the research process and its outcomes are culturally grounded, contextually relevant, and aligned with the values and priorities of the communities involved.

**Figure 3:**
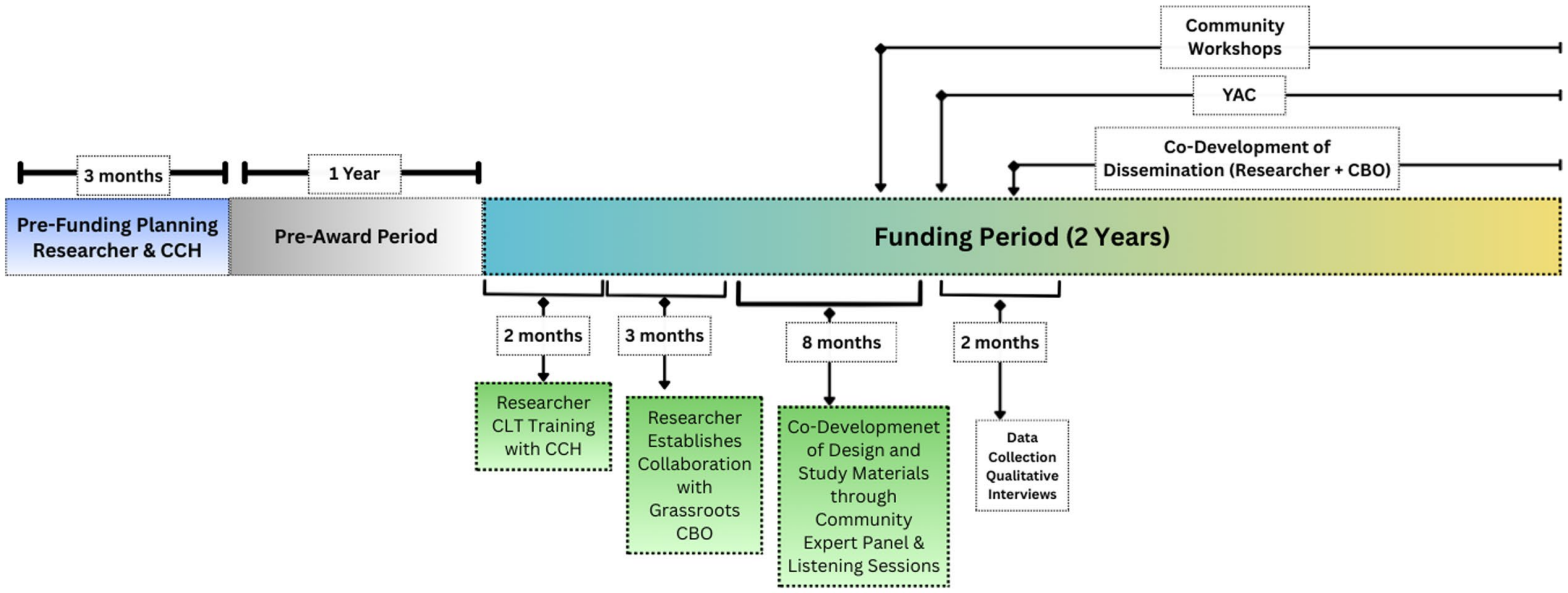
The timeline of our CEnR project.

Additionally, our partnership with the UCSD-CCH was instrumental in identifying the local, grassroots partner CBO, facilitating relationship building with the CBO, and establishing expectations for a mutually beneficial partnership including co-development of rules of engagement and promotion of equitable resource-sharing. Through this process, we established a partnership grounded in trust, transparency, and shared goals with the grassroots organization active in the selected study neighborhood site. Thus, the grassroots CBO brought deep insight into the local context and the trust of community members, a major community asset that would have been difficult to cultivate solely through UCSD-CCH, which, despite its extensive experience, does not operate at the grassroots level in this neighborhood. Working with both CBOs allowed us to integrate their unique strengths and perspectives on ways to incorporate and prioritize the health priorities of the study community. The timeline of our CEnR project is illustrated in Figure 3.

### 3.4 Interdisciplinary expertise

This project brings together an interdisciplinary team of diverse investigators with expertise in neurocognition, SU, and structural neighborhood determinants, recognizing that addressing complex public health issues like adolescents SU require integration across multiple domains of science and practice, meaningful community-engagement, and investigation of novel pathways and perspectives (52–55). Such interdisciplinary collaboration not only strengthens the scientific rigor of the project but also supports the mentorship and development of future scholars from all backgrounds, an essential component for workforce development for health research on adolescent development informed by the community as stakeholders (56).

The scientific team includes researchers from public health, neuroscience, psychology and clinical neuropsychology, each contributing distinct methodological and theoretical perspectives to bridge disciplines. Collectively, the team brings deep experience in neurocognitive development, addiction science, community-engaged methods, epidemiology, and longitudinal statistical modeling. This breadth is pivotal for understanding the multilevel pathways influencing adolescent SU, from individual cognitive vulnerabilities to NSRR. Additionally, our team’s partnerships include experienced university-based CBOs and emerging grassroots-level CBOs, ensuring that lived experience, local expertise, and structural inequities are centered in both the scientific questions and implementation strategies. Such partnerships not only strengthen the translational impact of the research but also build capacity for future community-driven studies (57).

## 4 Results

### 4.1 CEnR implementation

During the funding and study conceptualization phase, we integrated feedback from UCSD-CCH to ensure the research design prioritized community perspectives. Informed by these consultations, we selected COI 2.0 as a key variable from the ABCD dataset to serve both as a key predictor in the quantitative secondary data analyses and as a criterion for selecting the study site for primary qualitative data collection. This approach reflects our commitment to integrating evidence-based measures with community input throughout the research process.

Once the project secured funding, we engaged in 2-month planning meetings with the UCSD-CCH to identify a grassroots CBO in the urban neighborhood proposed for data collection. During these planning meetings, the UCSD-CCH provided the research team with training on implementation of the CLT framework in practice. UCSD-CCH, not only facilitated the introduction between the researcher and the grassroots CBO but also provided guidance and oversight in establishing expectations for a mutually beneficial researcher-CBO partnership. These multi-level partnerships allowed us to integrate community perspectives and assets to identify e and prioritize health outcomes that are important and relevant to the study community. The grassroots CBO brought deep insight into the local context, culture, knowledge, and trust of research among community members. UCSD-CCH, as a larger community-based organization, provided essential support to foster an equitable partnership between grassroots CBO and the research team, including co-development of rules of engagement, promotion of equitable resource-sharing, and building a mutually beneficial partnership grounded in trust, transparency, and shared goals.

Best practices in CEnR emphasize the importance of building trust with community members before inviting them into co-design processes or tool development (58, 59). Following this guidance, we engaged in 3-month planning meetings with the grassroots CBO to prioritize relationship-building, discovery of shared interests, establishing relevance and importance of research questions with community needs, setting mutual expectations and principles of collaboration. In alignment with our guidance framework and CLT principles, our study prioritizes capacity building and reciprocal benefit for community partners throughout the research process.

### 4.2 Community expert panel (CEP)

In partnership with the grassroots CBO, we formed a CEP comprised of four mothers of adolescents from the neighborhoods of study. The researchers and CEP met bi-weekly for 6 months to co-design the qualitative study and inform the approach of the quantitative study. Our community expert panel members were essential in guiding and informing our decisions and approach throughout all phases of the research project. Important discussions and insight led to the implementation of critical protocol adaptations to ensure safety and relevance of study outcomes to the community of study. For example, our CEP provided structured feedback on the interview protocols and study materials for the qualitative study to ensure the design and approach was accessible and culturally relevant for the community of study. Through this iterative process, substantial modifications were made to the study design. For example, the first version of the interview guide directly asked participants about exposure to substances in their neighborhoods. Community partners raised concerns that such direct, explicit questions could subject both participants and researchers to unwanted scrutiny. In response, the questions were revised to focus on neighborhood features such as areas that are “safe,” “enjoyable,” “unsafe,” or “places that get kids into trouble,” aligning with safety and relevance as priorities for community members. As suggested by the panel, these revised prompts and questions elicited meaningful insights related to substance use, even without explicit mention of it.

Our CEP members were instrumental in facilitating listening sessions with a larger group of mothers from the community. We held two listening sessions with 20–25 mothers to introduce our project and obtain critical feedback to ensure access and relevance of our study goals with the community. Through engagement with our CEP members and listening sessions, we were able to obtain and integrate critical changes in concepts and messaging into the final study design, recruitment strategy, engagement activities, study materials and community priorities on future dissemination of findings.

### 4.3 UCSD-CCH youth advisory council (YAC)

With support from the UCSD-CCH, we formed a study-specific group from members of the larger UCSD-CCH YAC Board. The UCSD-CCH YAC Program focuses on providing resources, leadership development opportunities and uplifting underserved youth voices and experiences. With this partnership, the study-specific YAC comprised of 15 members who participated in a 4-month internship to develop knowledge, skills, and receive training on research methods as well as content knowledge on theories of adolescent health. Guidance and input from YAC were essential components during the data collection phase of the qualitative study, contributing meaningfully to the refinement of recruitment strategies and compensation plans. Furthermore, YAC provided a critical adolescent perspective to inform our quantitative models and variable selection through member checking (60, 61). Nine of the YAC members then participated in a second 4-month internship to apply their training and knowledge of research through youth-led social media campaigns to support prevention and cessation of vaping and positive mental health among adolescents. The critical role of YAC in our research process provides evidence that youth involvement in research implementation leads to more thoughtful and impactful study designs (62). The investigative team will continue collaborating with the UCSD-CCH to sustain engagement with the YAC.

### 4.4 Continuous feedback loops

We implemented structured feedback loops to ensure that community input was not only thoughtfully solicited but also meaningfully incorporated at all stages of the project. A regular tempo of check-ins and follow-up advisory meetings with the UCSD-CCH, the grassroots CBO, community listening sessions, and YAC, created space for reflection, revision, and shared decision-making throughout the research process.

### 4.5 Opportunities for supporting research and health literacy for communities

From the very beginning of our engagement with the grassroot CBO, we prioritized research literacy training to demystify the research process and enhance community partners’ understanding of study design, data collection, ethical considerations, and the use of research findings. These sessions/meetings were tailored to ensure accessibility and relevance to reflect the sociocultural context of participating neighbourhoods. In partnership with the CBO, members of the research investigative team lead a series of community talks to present on key topics including, the purpose and process of research, consent, adolescent health behavior and the importance of research for advancing our understanding of prevention and intervention programs to support adolescent health. As part of our reciprocal engagement, we conducted health education workshops that addressed community-nominated topics, such as parenting, parent-youth communication, adolescent physical and mental health. The workshop topics were organized and conducted in collaboration with the grassroot CBO and incorporated culturally responsive materials to promote practical knowledge and well-being. Creating learning and training opportunities around the research process was an important step toward obtaining meaningful feedback and engagement from the community into the research process.

### 4.6 Research advocacy tools

To support sustained community engagement, we plan to continue to co-develop with our CEP and YAC advocacy tools such as infographics and one-page data summaries on adolescent health. These resources will empower community members to share findings with broader audiences, advocate for local policy changes, and amplify their voices in public forums and online platforms. The latter half of the internship aimed to equip youth with the skills needed to design, launch, and manage impactful social media campaigns focused on anti-vaping awareness and positive peer affirmations. Over the course of the program, participants engaged in eight 1-h group meetings, two 1.5-h public health seminars, and two individual meetings with the lab team. In addition to guided sessions, interns completed approximately 7 h of independent work to develop and implement their campaign strategies.

### 4.7 Support for future community-led research

Recognizing that true reciprocity includes fostering community autonomy, we offered technical assistance and mentorship to the CBO to support future community-led research proposals. This included guidance on research conceptualizations of community-led interventions and health priorities. As a result, a grant application was co-developed between the research investigators and the grassroots CBO to support a community-led intervention evaluation project focusing on well-being among parents/guardians of adolescents.

### 4.8 Future planned activities

The dissemination plan of the study findings will incorporate a strategy that ensures sharing of findings with the scientific community, community members, and policy makers. To ensure community engagement principles and equal translational opportunity, we will co-develop dissemination efforts that maximize accessibility, cultural relevance, and real-world impact.

Firstly, findings will be shared with the scientific audience through traditional academic channels, including: (i) peer-reviewed manuscripts in high-impact journals in public health, adolescent development, and SU, and (ii) presentations at national and local conferences.

Secondly, as part of our community-facing dissemination, to promote transparency and relevance for participants and their communities, we will: (i) co-produce plain-language briefs that summarize key findings and implications, distributed in print and digital formats. (ii) co-design infographics and social media materials tailored to adolescents and families, with visual storytelling to support wider reach and comprehension, (iii) co-host community town halls and workshops hosted in collaboration with grassroots partners to discuss results and gather feedback on interpretations and next steps, (iv) distribute materials in English and Spanish, formatted for low-literacy audiences when appropriate.

Finally, findings with potential policy implications will be translated into: (i) executive summaries for local and regional decision-makers in health, education, and youth services, (ii) briefing sessions or roundtable discussions with community coalitions and advisory boards to support systems-level action, (iii) collaboration with advocacy partners to align findings with ongoing legislative or funding initiatives related to adolescent well-being and neighborhood equal opportunity.

## 5 Discussion

The protocol outlines our study, which utilizes a CeNR, convergent mixed-methods design. This approach combines qualitative data collection with a secondary analysis of the large-scale longitudinal ABCD dataset to examine the neurocognitive mechanisms of SU among adolescents living in neighborhoods with limited opportunities and restricted access to resources. The integration of CEnR across both components of the research methods ensures that the inquiry is deeply grounded in the lived realities of adolescents with a variety of different lives experiences. While secondary datasets offer breadth, statistical power, and generalizability, they often fail to capture culturally specific risk and resilience factors or reflect community-defined priorities. By incorporating CeNR approach in variable selection, co-design of research tools, shared interpretation, and dissemination strategies, this study amplifies community voice and enhances the cultural relevance and validity of findings. The novelty lies in leveraging the strengths of both data modalities: large-scale quantitative analysis to detect multi-level patterns and mechanisms, and community-informed qualitative inquiry to contextualize those findings with depth and nuance. This hybrid approach not only addresses limitations inherent in secondary data but also advances equal health opportunity research by embedding community priorities into the frontiers of scientific investigation.

A mixed-methods design with CEnR approaches are key in understanding how neighborhood environments influence adolescents of all types of identities. Research demonstrates that neighborhood factors play a critical role in shaping how Hispanic adolescents define their identities (63, 64). Given that adolescents’ perceptions of their social environments shape their sense of self and behavioral choices, incorporating neighborhood factors may help explain both the underlying reasons for SU initiation, the extent of their engagement in SU, or if neighborhoods serve as a protective factor (65–67). Although limited research has examined the influence of neighborhood characteristics on SU among Hispanic adolescents, much of the existing mixed-methods research has primarily focused on neighborhood factors and Hispanic physical health (68–70). Scholars such as Pasco and White (63) have utilized mixed methods designs to explore how both researchers and Hispanic adolescents perceive neighborhood features and have investigated how these features have contributed to lived experiences. However, further research is needed to investigate how Hispanic adolescents themselves interpret their neighborhood environments and the sociocultural meanings they ascribe to their communities (65–67).

Large secondary datasets can greatly facilitate the examination of mechanisms that create and maintain health disparities among populations (7). Although CEnR is recognized as essential for promoting equal health opportunities, it is not commonly applied in studies that examine health disparities using secondary datasets. As a result, researchers are likely to miss out on the substantial opportunities to select variables, explore mechanisms and interpret results in ways that align with community-defined priorities and equal health opportunity goals. By integrating CEnR principles *post hoc*, researchers can revisit secondary datasets with a more critical and culturally attuned lens, examining variables through frameworks that prioritize resilience, structural determinants, and culturally specific protective factors, rather than deficit-based assumptions (1, 2, 13).

Furthermore, secondary datasets can be used to validate and scale up themes that emerge from qualitative data, enhancing generalizability while maintaining contextual integrity (23, 71). This approach enables a bidirectional translation between community narratives and population-level patterns, converging on actionable, equal opportunity-informed solutions. When combined with primary qualitative investigation and participatory dissemination strategies, large datasets can become powerful instruments for advocacy, structural reform, and culturally responsive public health interventions (14, 25).

### 5.1 Anticipated challenges and mitigation

Mistrust of academic institutions and perceived power asymmetries can undermine participation and partnership between researchers and communities, especially in communities with a history of research extraction of data or harm (2, 25). To address this, the study utilized a co-leadership model that emphasizes shared decision-making, transparency, and mutual respect throughout all phases of the research (24) By involving community partners in study design, recruitment, data interpretation, and dissemination, and ensuring equitable resource distribution, the research team aims to cultivate trust-driven, ethically grounded relationships that prioritize community autonomy and shared benefit (3, 18). While the feedback process was time-intensive, it was essential for ensuring that the research was ethically sound and grounded in the lived realities of the communities involved.

Convergent mixed-methods designs, especially those combining primary qualitative inquiry with secondary quantitative data, often face timing misalignments due to the iterative nature of community engagement and data governance requirements (71, 72). To mitigate this, the research team implemented a flexible scheduling framework that allows components to progress in parallel when appropriate, with built-in opportunities for reassessment and realignment. Flexibility ensures that community-informed qualitative data collection is not rushed and can unfold at a pace that honors relationship-building and iterative feedback without compromising alignment with the larger secondary dataset’s analytical timeline.

Sustained engagement with communities, especially those that are structurally disadvantaged, can be time and resource-intensive, resulting in fatigue and disengagement (58, 62). This study proactively addresses that risk by offering meaningful compensation, capacity-building opportunities, and continuous feedback loops. Community stakeholders received financial and educational recognition for their contributions, while results are shared in accessible formats that support their advocacy goals (25). While it is recommended to engage grassroots community partners from the very beginning of the study conceptualization (2), we utilized a multistage engagement strategy. Our pre-award engagement was with UCSD-CCH, an established organization with infrastructure to support uncompensated consultation. As a newer organization with limited infrastructure and experience in academic partnerships, the grassroots CBO was likely to be burdened by unfunded expectations during the pre-award period (73). Forming these focused partnerships with grassroots organizations and YAC post-award prevented premature demands on the time and capacity of multiple community members and organizations, particularly those with limited infrastructure. Second, it supported provisions for engagement that can be compensated, role-specific, and guided by mutually aligned goals (25). However, to facilitate meaningful CEnR, grant funders need to incorporate systems so that community partners can be compensated from the study conceptualization phase. This can support integration of CEnR approaches in the study design conceptualizations lead by advanced doctoral students and early career scientists, who may not have discretionary funds for community engagement in the pre-award phase.

### 5.2 Strengths and limitations

This protocol presents an innovative mixed-methods study that integrates CEnR approaches to address limitations in studying factors of risk and resilience for SU among Hispanic adolescents, a stigmatized topic among all populations. By incorporating community voices throughout both the secondary quantitative analysis and primary qualitative data collection, the study ensures that lived experiences remain central to the research process. In doing so, the protocol combines the methodological rigor and efficiency of mixed methods with the depth, relevance, and equal opportunity fostered through meaningful community engagement.

While the qualitative sample of Hispanic adolescents is small, its strength lies in capturing first-person rich narratives of neighborhood social factors that may not be evident through quantitative analyses of the ABCD dataset. Although neighborhood experiences can vary regionally, structural inequities and reduced neighborhood opportunities are pervasive across the U.S., especially in communities of color. As such, while some NSRR may be region-specific, we anticipate that many will generalize to adolescents living in similarly low-opportunity environments nationwide. The region-specific qualitative findings will be used to assess the broader applicability of identified NSRR across other ABCD sites. The San Diego ABCD site, with a large and diverse sample (~700 participants, ~65% Hispanic), presents an opportunity to conduct geographically informed analyses grounded in the qualitative data. However, the generalizability of the findings may be limited to urban contexts and may be most relevant to minoritized adolescents residing in neighborhoods with substantial representation of racial/ethnic minoritized populations.

Harmonizing secondary and primary data presents several methodological and practical challenges. Secondary datasets are often collected for different purposes, using standardized measures and broad sampling strategies, which may not align with the context-specific nature of primary qualitative data. Differences in the level of analysis (e.g., individual vs. neighborhood), data structure, timing of data collection, and population representation can complicate integration. Additionally, reconciling constructs across data sources, such as aligning youth-reported neighborhood experiences with census-derived indices, requires careful operationalization to ensure conceptual coherence. We acknowledge these challenges and create robust strategies that incorporate thoughtful integration through iterative analytic strategies, consultation with multidisciplinary experts, and engagement with community partners to enhance interpretability and relevance of findings across data sources.

## 6 Conclusion

This protocol reaffirms the importance of meaningful community engagement in advancing equitable research, particularly in studies aiming to improve health outcomes among communities that did not historically have equal opportunities. By integrating CEnR principles with a mixed-methods approach, this study offers a framework for centering community voices throughout the research process from conceptualization to dissemination. The protocol holds potential for guiding future research designs that seek to integrate community engagement and bridge primary qualitative inquiry with secondary quantitative data to yield more contextually grounded and actionable insights. Beyond academic contributions, the study aims to generate findings that are directly relevant to real-world clinical and public health practices, support community-driven policy advocacy, and ultimately improve health outcomes for adolescent populations of all backgrounds.
